# Cancer

**Published:** 1874-11

**Authors:** 


Dr. Benj. Rhett says: * * * I offer the above as my mite, believing that both the above remedies, and especially the chlorate of potass., have undoubted merit as therapeutic agents in treatment of cancerous growths; and may we not hope, that with the three agents, Marsden's arsenical mucilage, chlorate of potass, and bromine, that the poor sufferer's from cancer will no longer be told that "their cases are hopeless."—*Charleston Medical Journal and Review.*

